# Prior frequency guided diffusion model for limited angle (LA)-CBCT reconstruction

**DOI:** 10.1088/1361-6560/ad580d

**Published:** 2024-06-26

**Authors:** Jiacheng Xie, Hua-Chieh Shao, Yunxiang Li, You Zhang

**Affiliations:** 1 The Advanced Imaging and Informatics for Radiation Therapy (AIRT) Laboratory, The Medical Artificial Intelligence and Automation (MAIA) Laboratory, Department of Radiation Oncology, University of Texas Southwestern Medical Center, Dallas, TX 75390, United States of America

**Keywords:** cone-beam CT, diffusion model, limited angle, image reconstruction

## Abstract

*Objective.* Cone-beam computed tomography (CBCT) is widely used in image-guided radiotherapy. Reconstructing CBCTs from limited-angle acquisitions (LA-CBCT) is highly desired for improved imaging efficiency, dose reduction, and better mechanical clearance. LA-CBCT reconstruction, however, suffers from severe under-sampling artifacts, making it a highly ill-posed inverse problem. Diffusion models can generate data/images by reversing a data-noising process through learned data distributions; and can be incorporated as a denoiser/regularizer in LA-CBCT reconstruction. In this study, we developed a diffusion model-based framework, prior frequency-guided diffusion model (PFGDM), for robust and structure-preserving LA-CBCT reconstruction. *Approach.* PFGDM uses a conditioned diffusion model as a regularizer for LA-CBCT reconstruction, and the condition is based on high-frequency information extracted from patient-specific prior CT scans which provides a strong anatomical prior for LA-CBCT reconstruction. Specifically, we developed two variants of PFGDM (PFGDM-A and PFGDM-B) with different conditioning schemes. PFGDM-A applies the high-frequency CT information condition until a pre-optimized iteration step, and drops it afterwards to enable both similar and differing CT/CBCT anatomies to be reconstructed. PFGDM-B, on the other hand, continuously applies the prior CT information condition in every reconstruction step, while with a decaying mechanism, to gradually phase out the reconstruction guidance from the prior CT scans. The two variants of PFGDM were tested and compared with current available LA-CBCT reconstruction solutions, via metrics including peak signal-to-noise ratio (PSNR) and structural similarity index measure (SSIM). *Main results.* PFGDM outperformed all traditional and diffusion model-based methods. The mean(s.d.) PSNR/SSIM were 27.97(3.10)/0.949(0.027), 26.63(2.79)/0.937(0.029), and 23.81(2.25)/0.896(0.036) for PFGDM-A, and 28.20(1.28)/0.954(0.011), 26.68(1.04)/0.941(0.014), and 23.72(1.19)/0.894(0.034) for PFGDM-B, based on 120°, 90°, and 30° orthogonal-view scan angles respectively. In contrast, the PSNR/SSIM was 19.61(2.47)/0.807(0.048) for 30° for DiffusionMBIR, a diffusion-based method without prior CT conditioning. *Significance*. PFGDM reconstructs high-quality LA-CBCTs under very-limited gantry angles, allowing faster and more flexible CBCT scans with dose reductions.

## 1. Introduction

Cone-beam computed tomography (CBCT) has been widely applied to fields like radiotherapy for efficient and high-resolution volumetric imaging (Walter *et al* 2020). Limited-angle CBCT (LA-CBCT), which allows faster imaging speed, lower x-ray dose, and better mechanical clearance, has been investigated extensively (Zhang *et al* 2013a, 2013b, 2015b, Je *et al* 2014, Ren *et al* 2014). The analytical filtered back-projection (FBP)-based Feldkamp–Davis–Kress (FDK) (Feldkamp *et al* 1984) algorithm is widely used for CBCT reconstruction, which produces high-quality CBCT images given a complete set of projection data. However, given limited scan angles for LA-CBCT, FDK produces low-quality reconstructions with under-sampling artifacts and anatomical distortions. A well-established alternative for tomographic reconstruction is model-based iterative reconstruction (MBIR) (Katsura *et al* 2012, Liu 2014), which formulates the reconstruction as a 3D inverse problem and solves it by optimizing a physics-based data consistency model with additional regularization terms to remove the imaging artifacts induced by the limited projections. For regularization, the total variation (TV) penalty is widely used due to its edge-preserving and smoothing capabilities (Sidky and Pan 2008). MBIR or similar techniques have achieved substantial reconstruction improvement for sparsely sampled or low-dose projections (Katsura *et al* 2012, Pickhardt *et al* 2012, Chang *et al* 2013, Rui *et al* 2015). However, in the context of LA-CBCT reconstruction, these methods usually produce sub-optimal results given the lack of information from a full block of scan angles (Shu and Entezari 2022), which necessitates the use of stronger imaging priors to guide the reconstruction (Majee *et al* 2021). To introduce patient-specific priors, registration-driven approaches, which use limited-angle projections to solve LA-CBCTs via deforming previously acquired CT/CBCT images (Zhang *et al* 2013b, Ren *et al* 2014, Zhang 2021), have been introduced. However, compared with direct reconstruction-based methods, the registration-driven methods may fall short in solving non-deformation-induced anatomy changes (Zhang *et al* 2017a), and the imaging intensity variations between different imaging systems/protocols may impact the stability of such methods (Zhang *et al* 2015a). With the recent success of deep learning (DL) techniques, several works have been proposed for limited-angle/view image reconstruction. For instance, sinogram-based methods used generative adversarial networks (GANs) to predict full-angle/view projection data from limited-angle/view acquisitions, and then employed traditional methods like FDK to reconstruct artifact-free CBCTs from the predicted fully sampled data (Li *et al* 2019). Image-domain methods, on the other hand, employed convolutional neural networks (CNNs) to enhance image quality by directly mapping the artifacts-ridden reconstructions to corresponding high-quality images (Chen *et al* 2017, Gu and Ye 2017, Gupta *et al* 2018). Meanwhile, dual-domain methods such as DuDoNet (Lin *et al* 2019) trained CNNs in both sinogram space and image space to improve the image reconstruction quality and outperformed the former two strategies. However, such methods are often trained under a fixed under-sampling geometry/scenario, and may not generalize well to scenarios with flexible under-sampling patterns. Methods that combine neural networks with iterative physics-informed reconstruction models can often achieve better results and are more robust to scan scenario variations (Chen *et al* 2022, Hu *et al* 2022). These hybrid methods effectively leverage the power of neural networks in feeding prior information and serving as denoisers, and the proven records of physics-driven iterative reconstructions in solving model-based inverse problems. Different neural networks, including GAN, have been used as imaging priors to regularize model-based reconstructions, including LA-CBCT reconstruction (Yang *et al* 2020, Barutcu *et al* 2021, Zhou *et al* 2021, Gao *et al* 2023). However, such methods may still fail, especially for very small scan angles, as the priors provided by these neural networks are insufficient or unstable.

In summary, the main challenge of LA-CBCT reconstruction stems from the complete occlusion of a full block of scan angles, which is deemed a more difficult problem than sparse-view but full-angle reconstruction, and often results in severe undersampling artifacts and structural distortions. MBIR techniques, while improving image quality through optimization and regularization, are highly dependent on prior models and show large performance degradations when available viewing angles are small. Additionally, registration-based methods that rely on deforming patient prior anatomies struggle with non-deformable anatomical changes and inter-scan variations in imaging intensity. Recently, DL approaches, including sinogram-based and image-domain methods, have shown promise but often lack generalizability across different under-sampling scenarios. Hybrid methods combining neural networks with physics-driven models are more robust to different limited-angle scenarios, but still fall short in scenarios of very limited scan angles due to insufficient prior information encoded in the neural networks.

Recently, diffusion models were introduced into the inverse problems of image generation/reconstruction, and have been shown to outperform popular generative models such as GAN (Dhariwal and Nichol 2021). Diffusion models operate through forward and backward diffusion steps (Ho *et al* 2020). In the forward process, a diffusion model gradually adds noise to an image, transforming the data into a Gaussian noise distribution. Starting from the noise, the model then iteratively reduces the noise, through a reverse diffusion process which is trained to reconstruct the original image or generate new samples from the learned data distribution. In the field of medical imaging, score-based diffusion models (Song and Ermon 2019, 2020, Song *et al* 2020, 2021) are a class of methods that achieve state-of-the-art (SOTA) performance in image generation. It provides a powerful way to learn population-based imaging statistics and distributions and can serve as strong imaging priors in medical imaging studies (Song *et al* 2021, Chung and Ye 2022). In terms of limited-angle CT (LA-CT) reconstruction, DiffusionMBIR (Chung *et al* 2023) and DOLCE (Liu *et al* 2023) are among the latest diffusion model-based methods that showed superior performance over the previous methods. By embedding the diffusion model as a denoiser in an alternating direction method of multipliers (ADMMs)-based iterative reconstruction framework, DiffusionMBIR uses the prior distribution learned in the diffusion model to enhance the LA-CT image quality. DOLCE (Liu *et al* 2023), on the other hand, similarly used the diffusion model to regularize an iterative model-based reconstruction, while conditioning the diffusion model with additional LA-CTs reconstructed by regularized least squares or FBP techniques. However, both the DOLCE technique and the DiffusionMBIR technique were developed for CT reconstruction using parallel beam geometries. A clinically realistic cone-beam imaging geometry can be more challenging for both methods. In addition, although both methods showed improved results in limited-angle reconstruction, it is still challenging to reconstruct high-quality and accurate images using relatively small gantry angles (i.e. $\theta {\text{ }} \unicode{x2A7D} 90^\circ $) due to the lack of patient-specific information.

To further explore the potential of diffusion model-driven reconstruction, we proposed a prior frequency-guided diffusion model (PFGDM) for LA-CBCT reconstruction. PFGDM is built upon the findings of our recent work on zero-shot image translation (Li *et al* 2023), which observed that a diffusion model conditioned on image-domain high-frequency information can better retain the anatomical details within the images. For source and target image domains of the same or similar underlying anatomy (for instance, CBCT and CT image domains of the same patient), the high-frequency information from one source image domain can be applied as patient-specific priors to condition the diffusion model for structural preservation during source-to-target domain image translation. In the context of radiotherapy, it is almost certain that patient-specific CTs of relatively high quality will be acquired for treatment simulation/planning before the following CBCT acquisitions for treatment guidance. Such CT information can serve a strong anatomical prior to condition the reconstruction of daily LA-CBCTs. Inspired by these observations, we developed PFGDM for LA-CBCT reconstruction, which is conditioned on high-frequency information extracted from patient-specific prior CT scans. However, although the anatomical structures are expected to be similar between CT/CBCT, they may not be exactly the same due to daily anatomical variations. To allow the reconstruction of CBCT anatomical structures that are different from those of CT, we proposed two ways of introducing the prior CT condition to the reconstruction: (1) PFGDM-A, which uses a condition-dropping mechanism to drop the prior CT condition after a preset step number, and continue reconstructing the CT/CBCT anatomical differences in the remaining reconstruction steps. (2) PFGDM-B, which continuously applies the conditional input in every reconstruction step. However, in each step, an increasing high-pass threshold is applied to the prior CT to gradually remove the edge information of the anatomical structures and phase out the guidance from the prior CT.

In this study, we evaluated the two variants of PFGDM and compared them with the other methods for LA-CBCT reconstructions across a range of limited angles ($\theta \in 30^\circ ,90^\circ ,120^\circ $) and three anatomical sites (lung, pelvis, and head and neck). We also evaluated the PFGDM’s potential on CBCT reconstruction using extremely limited scan angles ($2^\circ $ to $10^\circ $). Trained as a single model, PFGDM achieves SOTA performance across all scenarios, making it a highly promising tool for clinical applications.

## 2. Materials and methods

### 2.1. Background of LA-CBCT reconstruction and related methods

#### 2.1.1. Inverse problems

Reconstructing LA-CBCT can be formulated as a linear inverse problem:

\begin{align*}{\boldsymbol{y}} = {\boldsymbol{Ax}} + {\boldsymbol{n}},\end{align*}

where ${\boldsymbol{x}} \in {\mathbb{R}^n}$ is the image to be reconstructed, ${\boldsymbol{y}} \in {\mathbb{R}^m}$ is the measurement (i.e. sinogram), ${\boldsymbol{A}} \in {\mathbb{R}^{m \times n}}$ is the measurement system matrix, and ${\boldsymbol{n}}$ is the measurement noise in the system. For CBCT reconstruction, a standard optimization-based approach usually iteratively estimates ${\boldsymbol{x}}$ from the measurement ${\boldsymbol{y}}$ with additional TV regularization:

\begin{align*}{\boldsymbol{x}^*} = \arg \mathop {\min }\limits_{\boldsymbol{x}} \left( {\frac{1}{2}\left\| {\boldsymbol{y - Ax}} \right\|_2^2 + {{\left\| {\boldsymbol{Dx}} \right\|}_1}} \right),\end{align*}

where ${\text{ }}{\boldsymbol{D}}: = {[{\boldsymbol{D_x}},{\boldsymbol{D_y}},{\boldsymbol{D_z}}]^{\boldsymbol{T}}}$ represents the finite difference operator in each axis. Minimization of equation (2) can be performed with optimization algorithms such as ADMM. Reconstructing ${\boldsymbol{x}}$ from ${\boldsymbol{y}}$ is highly ill-posed for LA-CBCT, thus additional assumptions/priors on ${\boldsymbol{x}}$ are often needed.

#### 2.1.2. Score-based diffusion models

Score-based diffusion models (Song *et al* 2020) are a type of generative models that simulate the generation of data/images by reversing a data-noising process, which can be incorporated as a denoiser in inverse problems. Consider a stochastic process with the time variable $t \in \left[ {0,1} \right]$, the noise-corrupted data/image ${\boldsymbol{x}}$ can be represented as ${\boldsymbol{x}}\left( t \right) = {\boldsymbol{x}_t}$. Suppose the data is sampled from a distribution $p\left( {\boldsymbol{x}} \right)$ for ${\boldsymbol{x}}\left( 0 \right)$, as time progresses $\left( {{\text{ }}t = 1{\text{ }}} \right)$, the data distribution ${\boldsymbol{x}}\left( 1 \right)$ approaches a Gaussian distribution. The evolution of the data distribution over time is described by a stochastic differential equation (SDE):

\begin{align*}{\text{d}}{\boldsymbol{x}} = {\boldsymbol{f}}\left( {\boldsymbol{x},t} \right){\text{d}}t + g\left( t \right){\text{d}}{\boldsymbol{w}},\end{align*}

where ${\boldsymbol{f}}\left( {\boldsymbol{x},t} \right):{\mathbb{R}^{n \times 1}} \mapsto {\mathbb{R}^n}$ is a drift function, $g\left( t \right):\mathbb{R} \mapsto \mathbb{R}$ is a scalar diffusion function, and $\boldsymbol{w}$ denotes the $n$-dimensional standard Brownian motion (Särkkä and Solin 2019). For a specific case of SDE: variance exploding SDE (VE-SDE) (Song *et al* 2020), when discretizing the noise-adding process into $N$ steps (indexed by $i$), the perturbation to the distribution of state ${\boldsymbol{x}_i}$ can be represented by the following Markov chain:

\begin{align*}{\boldsymbol{x}_i} = {\boldsymbol{x}_{i - 1}} + \sqrt {\sigma _i^2 - \sigma _{i - 1}^2} {\boldsymbol{z}_{i - 1}},\quad i = 1, \cdots ,N,\end{align*}

where ${\boldsymbol{z}_{i - 1}}\sim \mathcal{N}\left( {0,{\boldsymbol{ I}}} \right)$ represents Gaussian noise with mean zero and identity covariance matrix added at each step of the Markov chain to perturb the state ${\boldsymbol{x}_i}$, ${\sigma _i}$ represents a sequence of positive noise scales such that ${\sigma _{\min }} = {\sigma _1} &lt; {\sigma _2} &lt; \cdots &lt; {\sigma _N} = {\sigma _{\max }}$ and we introduced ${\sigma _0} = 0$ to simplify the notation. Let ${\boldsymbol{x}}\left( {\frac{i}{N}} \right) = {\boldsymbol{x}_i}$, $\sigma \left( {\frac{i}{N}} \right) = {\sigma _i}$, and ${\boldsymbol{z}}\left( {\frac{i}{N}} \right) = {\boldsymbol{z}_i}$, for $i = 1,{\text{ }}2, \cdots ,N$. As $N \mapsto \infty $, $\left\{ \sigma \right\}_{i = 1}^N$ becomes a continuous function $\sigma \left( t \right)$, ${\boldsymbol{z}_i}$ becomes $z\left( t \right)$, and the Markov chain becomes a continuous stochastic process $\left\{ {\boldsymbol{x}\left( t \right)} \right\}_{t = 0}^1$ indexed by the time variable $t$. Equation (4) can then be rewritten as follows with $\Delta t = \frac{1}{N}$ and $t \in \left\{ {0,\frac{1}{N}, \cdots ,\frac{{N - 1}}{N}} \right\}$ (Song *et al* 2020):

\begin{align*}{\boldsymbol{x}}\left( {t + \Delta t} \right) = {\boldsymbol{x}}\left( t \right) + \sqrt {{\sigma ^2}\left( {t + \Delta t} \right) - {\sigma ^2}\left( t \right)} \boldsymbol{z}\left( t \right) \approx {\boldsymbol{x}}\left( t \right) + \sqrt {\frac{{{\text{d}}\left[ {{\sigma ^2}\left( t \right)} \right]}}{{{\text{d}}t}}} \Delta t\boldsymbol{z}\left( t \right),\end{align*}

where the approximate equality holds when $\Delta t \ll 1$. In the limit of $\Delta t \mapsto 0$, the SDE simplifies to the Brownian motion (Song *et al* 2020):

\begin{align*}{\text{d}}{\boldsymbol{x}} = \sqrt {\frac{{{\text{d}}\left[ {{\sigma ^2}\left( t \right)} \right]}}{{{\text{d}}t}}} {\text{d}}{\boldsymbol{w}}.\end{align*}

The data evolution in VE-SDE is characterized by a constant mean with increasing Gaussian noise, eventually leading to pure Gaussian noise. By applying Anderson’s theorem (Anderson 1982), the reverse of this SDE, which forms the basis of the generative process in score-based diffusion models, is defined as (Song *et al* 2020):

\begin{align*}{\text{d}}{\overline{\boldsymbol{ x}}} = - \frac{{{\text{d}}\left[ {{{{\sigma }}^2}\left( t \right)} \right]}}{{{\text{d}}t}}{\nabla _{{\boldsymbol{x}_t}}}\log p\left( {{\boldsymbol{x}_t}} \right){\text{d}}t + \sqrt {\frac{{{\text{d}}\left[ {{\sigma ^2}\left( t \right)} \right]}}{{{\text{d}}t}}} {\text{d}}\overline {\boldsymbol{w}} ,\end{align*}

where ${\nabla _{{\boldsymbol{x}_t}}}\log p\left( {{\boldsymbol{x}_t}} \right)$ is the score function, representing the gradient of the log probability with respect to the data. To solve the reverse-time SDE that defines the generative process of the diffusion model, we adopted the denoising score matching framework previously developed in other works (Vincent 2011, Song *et al* 2020) to train the neural network formulated as:

\begin{align*}\mathop {\min }\limits_{{\theta }} {E_{t,{\boldsymbol{x}}\left( t \right)}}\left[ {{{\lambda }}\left( t \right)||{\boldsymbol{s}_{{\theta }}}\left( {\boldsymbol{x}\left( t \right),t} \right) - {\nabla _{{\boldsymbol{x}_t}}}\log p\left( {\boldsymbol{x}\left( t \right){\text{|}}{\boldsymbol{x}}\left( 0 \right)} \right)||_2^2} \right],\end{align*}

where ${\boldsymbol{s}_\theta }\left( {\boldsymbol{x}\left( t \right),t} \right):{\mathbb{R}^{n \times 1}} \mapsto {\mathbb{R}^n}$ is a time-dependent neural network and $\lambda \left( t \right)$ is the weighting function. Ultimately, given this robust training schema (Chung *et al* 2023), ${\boldsymbol{s}_{{{{\theta }}^{\text{*}}}}}\left( {\boldsymbol{x}\left( t \right),t} \right) \simeq {\nabla _{{\boldsymbol{x}_t}}}\log p\left( {{\boldsymbol{x}_t}} \right)$ can be established. Hence, the reverse diffusion process is approximated by:

\begin{align*}{\text{d}}\overline {\boldsymbol{x}} \simeq - \frac{{{\text{d}}\left[ {{{{\sigma }}^2}\left( t \right)} \right]}}{{\text{d}t}}{{\boldsymbol{s}}_{{{{\theta }}^{\text{*}}}}}\left( {{\boldsymbol{x}}\left( t \right),t} \right){\text{d}}t + \sqrt {\frac{{{\text{d}}\left[ {{{{\sigma }}^2}\left( t \right)} \right]}}{{{\text{d}}t}}} {\text{d}}\overline {\boldsymbol{w}} .\end{align*}

To solve equation (9), one can employ methods such as the predictor–corrector (PC) sampler (Song *et al* 2020), which involves discretizing the time interval from 0 to 1 into $N$ equal steps. It allows the numerical integration of the reverse diffusion process to generate samples from the target distribution.

#### 2.1.3. Diffusion model in inverse problems

Training diffusion models involves solving the reverse SDE with the approximated score function defined in equation (9), which can be viewed as sampling from the prior distribution $p\left( {\boldsymbol{x}} \right)$. To solve inverse problems, we need to sample from the posterior distribution $p({\boldsymbol{x|y}})$. Based on the Bayes’ rule:

\begin{align*}p\left( {\boldsymbol{x}{\text{|}}{\boldsymbol{y}}} \right) = \frac{{p\left(\boldsymbol{ x} \right)p\left( {\boldsymbol{y}{\text{|}}{\boldsymbol{x}}} \right)}}{{p\left( \boldsymbol{y} \right)}}.\end{align*}

DiffusionMBIR (Chung *et al* 2023) is the one of the representative works that introduced diffusion models into solving inverse problems of reconstructing LA-CT images. In DiffusionMBIR’s framework, given the definition in equation (1), the inverse problem was solved using the diffusion prior in equation (9) with the Bayes’ rule, leading to:

\begin{align*}{\nabla _{\boldsymbol{x_t}}}\log p\left( {\boldsymbol{x_t}{\text{|}}{\boldsymbol{y}}} \right)&amp; = {\nabla _{\boldsymbol{x_t}}}\log p\left( {\boldsymbol{x_t}} \right) + {\nabla _{\boldsymbol{x_t}}}\log p\left( {\boldsymbol{y}{\text{|}}{\boldsymbol{x_t}}} \right)\nonumber\\ &amp; \simeq {\boldsymbol{s}_{{{{\theta }}^{\text{*}}}}}\left( {\boldsymbol{x}\left( t \right),t} \right) + {\nabla _{\boldsymbol{x_t}}}\log p\left( {\boldsymbol{y}{\text{|}}{\boldsymbol{x}_t}} \right).\end{align*}

To incorporate equation (11), the existing literature (Song *et al* 2020, Chung *et al* 2022) defined the update step as:

\begin{align*}{\boldsymbol{x}}^\prime_{t - 1}&amp; \leftarrow {\text{Solve}}\left( {{\boldsymbol{x}_{t - 1}},{\boldsymbol{s}_{{{{\theta }}^*}}}} \right),\end{align*}

\begin{align*}{\boldsymbol{x}_{t}}&amp; \leftarrow {\mathcal{P}_{\left\{ {\boldsymbol{x}{\text{|}}{\boldsymbol{y}} = {\boldsymbol{Ax}} + {\boldsymbol{n}}} \right\}}}{( {\boldsymbol{x}^\prime_{t - 1}})},\end{align*}

where ${\text{Solve}}$ denotes the reverse-SDE solver of equation (9), and ${{\mathcal{P}}_C}$ denotes the projection operator to the set $C = \left\{ {\boldsymbol{x}}{\text{|}}{\boldsymbol{y}} = {\boldsymbol{Ax}} + {\boldsymbol{n}} \right\}$.

#### 2.1.4. High-frequency information extraction

To extract the high-frequency information from prior CT images for PFGDM reconstruction, we used the Sobel operator (Kanopoulos *et al* 1988), a discrete differential operator. Specifically, we convolved the CT image $C{T_{{\text{prior}}}}$ with horizontal and vertical Sobel filter kernels, denoted as ${K_x}$ and ${K_y}$, respectively, resulting in two filtered images, ${G_x}$ and ${G_y}$. The gradient magnitude is obtained by combining the resulting gradient approximations:

\begin{align*}{G_x}&amp; = {K_x}{\text{*}}C{T_{\text{prior}}},\quad {G_y} = {K_y}{\text{*}}C{T_{\text{prior}}},\end{align*}

\begin{align*}{i_{{\text{mag}}}}&amp; = \sqrt {G_x^2 + G_y^2} .\end{align*}

The high-frequency information $H$ can then be extracted through applying a threshold $\eta $:

\begin{align*}{H_\eta } = \left\{ \begin{array}{*{20}{lc}} {{i_{{\text{mag}}}}\left( {x,y} \right)} &amp;{{i_{{\text{mag}}}}\left( {x,y} \right) \unicode{x2A7E} \eta} \\ {0}&amp; {{i_{{\text{mag}}}}\left( {x,y} \right) &lt; \eta } \end{array}\right..\end{align*}

### 2.2. The proposed method: PFGDM

**Figure 1.**
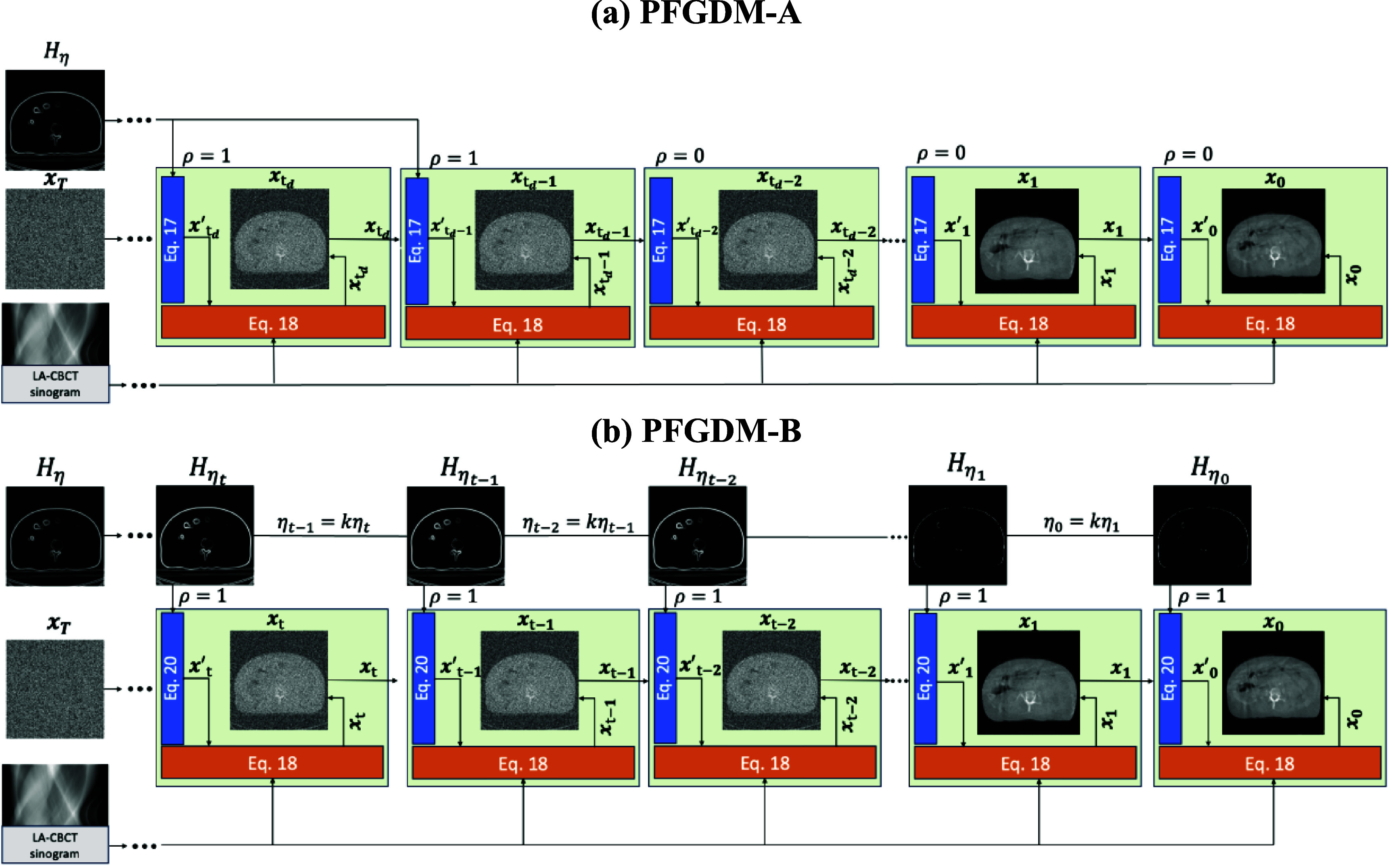
Overview of the two PFGDM variants: PFGDM-A and PFGDM-B. Starting from the Gaussian noise ${\boldsymbol{x}_T}$, in each SDE step of the diffusion model (blue box), we sample a LA-CBCT image ${\boldsymbol{x}^{\prime}_t}$ from the posterior by applying the reverse diffusion that is conditioned on the high-frequency prior CT information. Concurrent with the diffusion model, the LA-CBCT image is also iteratively updated via ADMM based on the given LA-CBCT sinogram (orange box), until convergence. (a) For PFGDM-A, the conditional input ${H_\eta }$ remains unchanged during reconstruction and is dropped after ${\boldsymbol{x}_{{t_d} - 1}}$. (b) For PFGDM-B, the conditional input ${H_{{\eta _t}}}$ is continuously introduced along the process while dynamically updated to gradually phase out the edge information of the prior CT.

An overview of the PFGDM method and its two variants is shown in figure 1. In general, PFGDM reconstructs CBCTs from LA-CBCT projections with the guidance of a conditional diffusion model, where the condition $H$ is obtained by applying the Sobel operatort to the prior CT (equation (1)). Solving the inverse problem with the diffusion model involves solving the reverse SDE with the approximated score function defined in equation (9), which can be viewed as sampling from the conditional posterior distribution $p({\boldsymbol{x}}|{\boldsymbol{y}},{\text{ }}H)$. To avoid the excessive computational hardware/power required by 3D diffusion models, we use the 2D diffusion model for 3D LA-CBCT reconstruction. Inspired by DiffusionMBIR (Chung *et al* 2023), we applied the diffusion model slice by slice while adding TV regularization along the *z*-axis (slice direction) to regularize the inter-dependency between slices. Specifically, equation (12) is enforced by applying a 2D diffusion model-based regularizer slice-by-slice, while equation (13) is realized through ADMM-based iterative reconstruction with TV regularization.

Thus, the solution of PFGDM-A can be formulated as:

\begin{align*}{\boldsymbol{x}}^\prime_{t - 1}&amp; \leftarrow {\text{Solve}}\left( {{\boldsymbol{x}_t},{\boldsymbol{s}_{{{{\theta }}^{\text{*}}}}},\rho {H_\eta }} \right),\end{align*}

\begin{align*}{\boldsymbol{x}_{t - 1}}&amp; \leftarrow \arg \mathop {\min }\limits_{x^{^{\prime}}_{t - 1}} \left( {\frac{1}{2}\left\| {\boldsymbol{y - Ax}^\prime_{t - 1}} \right\|_2^2 + {{\left\| {{\boldsymbol{D_z}}{\boldsymbol{x}}^\prime_{t - 1}} \right\|}_1}} \right),\end{align*}

where ${\left\| {{D}_{\boldsymbol{z}}{\boldsymbol{x}}^\prime_{t - 1}} \right\|_1}$ represents the L1 norm of the finite differences of $x^\prime_{t - 1}$ along the *z*-axis only, and ${H_\eta }$ denotes the Sobel-filtered prior CT information. As the reconstruction is coupled with the reverse diffusion process, the iteration here was denoted in a descending order to match the notation norm, i.e. from *T* to 0. Specifically, equation (17) equals to denoising each slice in parallel by the trained score-based diffusion model under the conditional input ${H_\eta }$. Here ${\text{Solve}}$ refers to the PC solver that alternates between the numerical SDE solver and Monte Carlo Markov chain steps, which shows outstanding performance according to Song’s work (Song *et al* 2020). Equation (18) applies the $\boldsymbol{z}$-directional TV prior and enforces data fidelity. Notably, $\rho $ is a hyper-parameter that determines whether the conditional input ${H_\eta }$ exists or not, which controls the condition-dropping mechanism. The conditional input ${H_\eta }$ of PFGDM-A remains unchanged during the reconstruction process, while $\rho $ is changed from 1 to $0$ after a pre-set step number to stop introducing the condition.

For PFGDM-B, the corresponding formulation for the conditional diffusion is:

\begin{align*}{H_{{\eta _{t - 1}}}}&amp; = \left\{ \begin{array}{@{}ll} {{i_{{\text{mag}}}}} &amp; {{i_{{\text{mag}}}} \unicode{x2A7E} {\eta _{t - 1}}} \\ {0} &amp; {{i_{{\text{mag}}}} &lt; {\eta _{t - 1}}} \end{array},{\text{ }}{\eta _{t - 1}} = \frac{t}{{t - 1}}{\eta _t}\right.\end{align*}

\begin{align*}{\boldsymbol{x}}^\prime_{t - 1}&amp; \leftarrow {\text{Solve}}\left( {{\boldsymbol{x}_t},{\boldsymbol{s}_{{{{\theta }}^{\text{*}}}}},\rho {H_{{\eta _{t - 1}}}}} \right),\end{align*}

where ${\eta _{i - 1}} = k{\eta _i}$ defines the adaptive high-pass threshold. In this reconstruction schema, the increasing high-frequency threshold gradually phases out the edge information along the reconstruction process. Compared with PFGDM-A, PFGDM-B does not need to specifically optimize the step number where the condition is dropped. Simiarly, the iteration number was denoted in a descending order here to match the reverse diffusion process.

### 2.3. Experimental setup

#### 2.3.1. Datasets and pre-processing

**Figure 2.**
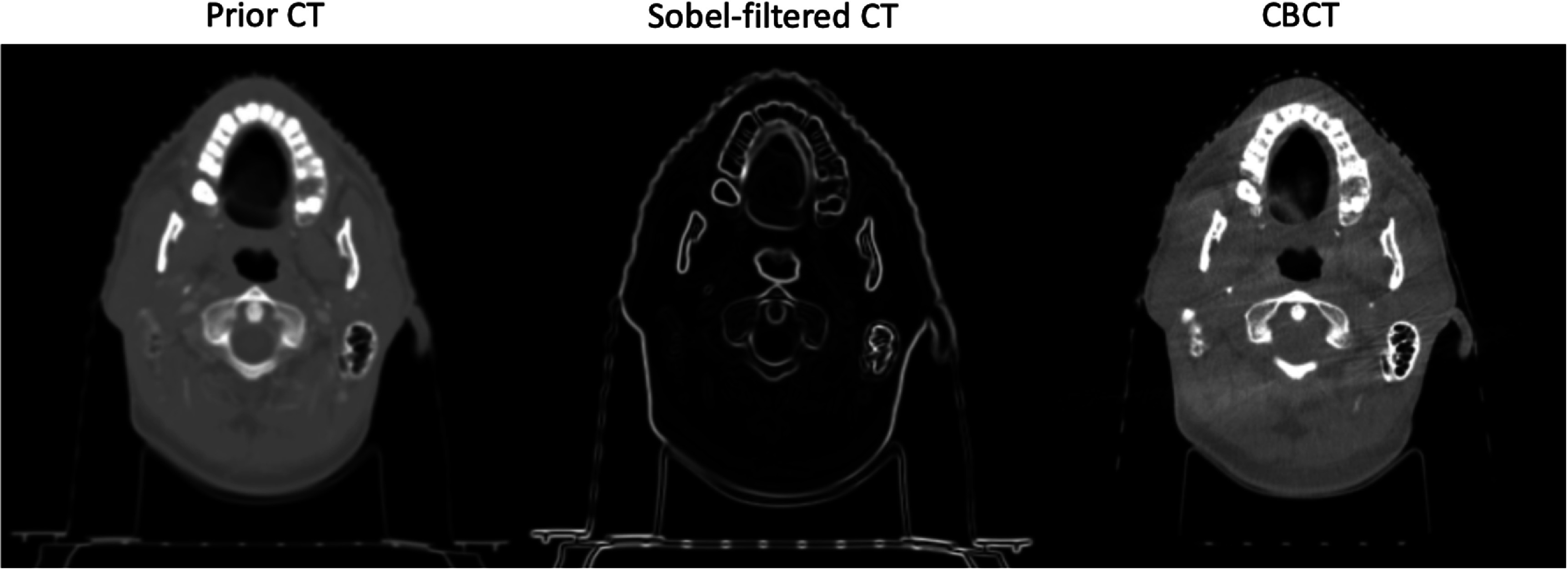
Samples from the head and neck dataset: the prior CT scan (left), the extracted high-frequency information of the prior CT scan (middle), and the CBCT scan (right) of the same patient.

To train the conditional score-based diffusion model, we used an in-house dataset consisting of ∼3000 CBCT slices from 30 patients of three anatomical sites: head and neck, lung, and pelvis. All CBCT slices were resized to ${\text{ }}256\, \times \,256$. Each CBCT volume was then intercepted by a Hounsfield unit range of [−1000, 1000] and linearly normalized to [0, 1]. The high-frequency information of each CBCT slice is extracted using the Sobel filter function from the Scipy package (Virtanen *et al* 2020). For testing, we used CT and CBCT scans from 12 different patients of the three anatomical sites (4 patients for each site). The CT scans were rigidly registered to the corresponding CBCT images using the open-source software package Elastix (Klein *et al* 2010). All CT and CBCT images in the testing set were similarly resized, intercepted, and linearly normalized as the training data. The high-frequency information of the prior CTs was extracted using the Sobel filter as well. Figure 2 shows a set of sample images from the head and neck dataset.

Cone-beam x-ray projections were simulated on-the-fly using the Operator Discretization Library package (Kohr and Adler 2017) during the test-time LA-CBCT reconstruction. We simulated limited-angle acquisitions from a single direction (single-view) or orthogonal directions (orthogonal-view), for the latter has shown better sampling efficiency under the same total scan angle as in previous works (Zhang *et al* 2013b). For single-view projections, the projection angles have ranges of $\left[ {0^\circ ,30^\circ } \right]$, $\left[ {0^\circ ,90^\circ } \right]$, and $\left[ {0^\circ ,120^\circ } \right]$ for $30^\circ $, $90^\circ $, and $120^\circ $ scan angle scenarios, respectively. For orthogonal-view projections, the projection angles have ranges of $\left( {\left[ {0^\circ ,15^\circ } \right],\left[ {90^\circ ,105^\circ } \right]} \right)$ for $30^\circ $, $\left( {\left[ {0^\circ ,45^\circ } \right],\left[ {90^\circ ,135^\circ } \right]} \right)$ for $90^\circ $, and $\left( {\left[ {0^\circ ,60^\circ } \right],\left[ {90^\circ ,150^\circ } \right]} \right)$ for $120^\circ $ scan angle scenarios. The projections were simulated of 256 × 256 pixels, with each pixel measuring 1.552 × 1.552 mm^2^ in dimension. The distance from the source to the detector and to the isocenter was set to 1500 mm and 1000 mm, respectively. The angular sampling density was 1 projection per degree.

#### 2.3.2. Training and evaluation schemes

In the training process, the Sobel-filtered CBCT slices were fed as optional conditional input to guide the diffusion model (no CT information needed). Specifically, for PFGDM-A, during the diffusion model training, we used a random number generator to guide the random introduction of this extracted high-frequency information with a probability of 0.5 during each reverse diffusion step. In detail, the model has equal chances of getting high-frequency information and −1 tensors of the same shape as conditional input. Thus, the model is trained to fit scenarios with and without the conditional input, allowing us to drop the condition when appropriate during the test time to reconstruct CT/CBCT differences. For PFGDM-B, the extracted high-frequency information was consistently applied during each reverse diffusion step, while filtered by a random high-pass threshold that ranges from its minimum to maximum values. The two models were implemented using the PyTorch 2.1.2 library with CUDA 12.1 and trained on an NVIDIA RTX 3090 graphic processing unit (GPU) card separately for one week. All test-time reconstructions were run on an NVIDIA Tesla V100 GPU card.

We reconstructed LA-CBCTs using five different methods (FDK, ADMM-TV, DOLCE, DiffusionMBIR, and PFGDM), based on the limited-angle acquisition scenarios simulated in section 2.3.1. We adapted DOLCE (Liu *et al* 2023) and DiffusionMBIR (Chung *et al* 2023) in this study by modifying the projection geometry from parallel beam to cone beam. Compared with DiffusionMBIR and PFGDM, different DOLCE models need to be retrained for different limited-angle acquisition geometries. To compare the image reconstruction performance of each method, we measured the accuracy of the reconstructed images using PSNR (peak signal-to-noise ratio) and structural similarity index measure (SSIM) (Wang *et al* 2004) by comparing with the ‘ground-truth’ CBCT images. To further evaluate the potential of PFGDM, we compared the reconstruction results of PFGDM-A and PFGDM-B under extremely limited scan angles (2°, 4°, 8°, and 10°).

The x-ray projections already included some noise as they are projected from noise-contained CBCT images. For a further noise study, we added both x-ray photon and electronic noises to the simulated projections. The photon noise was modeled as a Poisson distribution with a mean of 10^5^ and 10^6^ photons for each x-ray detector pixel, and the electronic noise was modeled as a Gaussian distribution with a variance of 10 photons (Wang and Gu 2013, Zhang *et al* 2013b).

As aforementioned, the CBCT-CT pairs in our study were rigidly registered to bring the to-be-reconstructed CBCT closer to the CT prior. Clinically, the initial alignment can also be performed via 2D x-ray projection-based matching or 2D/3D registration. To further evaluate the impact of misaligned CT/CBCT on the reconstruction accuracy, we spatially shifted the registered CT image by magnitudes of 1 mm, 2 mm, or 4 mm along one random in-plane direction (anterior–posterior or lateral) for each test sample and assessed the reconstruction results.

In addition, we performed two ablation studies by evaluating the contribution of the prior CT guidance and the contribution of the ADMM updating step, respectively. We first tested reconstructing CBCTs in the absence of the prior CT information guidance. Our PFGDM model, PFGDM-A, is trained to work both with and without prior information to fit the flexible condition-dropping mechanism. Thus, PFGDM-A can still be used when the prior information is not available. The scenario of missing prior information can be considered an extreme case of PFGDM-A, where the prior condition is dropped at step 1 of the reconstruction. Besides, we tested generating CBCTs without the ADMM updating step. The ADMM updating step is designed to incorporate the limited-angle projection information into solving the inverse problem. By removing the limited-angle information from the reconstruction process, the diffusion model is conditioned only on the CT edge information.

## 3. Results

### 3.1. Hyper-parameter analysis for PFGDM-A

**Figure 3.**
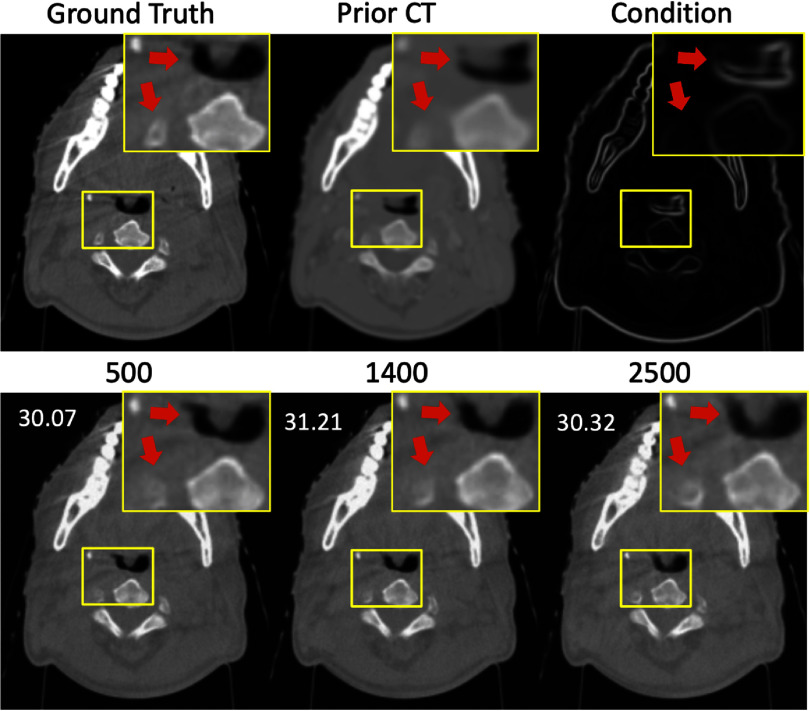
Comparison between reconstructed LA-CBCTs, with the high-frequency prior CT condition dropped at different iteration steps (500, 1400, and 2500) for PFGDM-A. The reconstructions are based on an orthogonal-view 90° scan angle (45° for each view), using a total of 3000 iteration steps. PSNR scores measured against the ‘ground-truth’ fully-sampled CBCT are indicated at the top-left corner.

As aforementioned, in equation (17) the hyper-parameter $\rho $ controls the presence of the conditional input during reconstruction for PFGDM-A. With $\rho $, we can choose to stop introducing the high-frequency information after a preset number of steps during reconstruction, to allow differing CT/CBCT anatomies to be better reconstructed. Figure 3 shows reconstructed LA-CBCTs (second row), with the condition extracted from the prior CT dropped at various iteration steps. It can be seen that dropping the condition at an intermediate step (1400) allows the LA-CBCT to inherit similar anatomical features from the prior CT (1400 vs. 500), while better correcting the anatomical mismatches (1400 vs. 2500).

**Figure 4.**
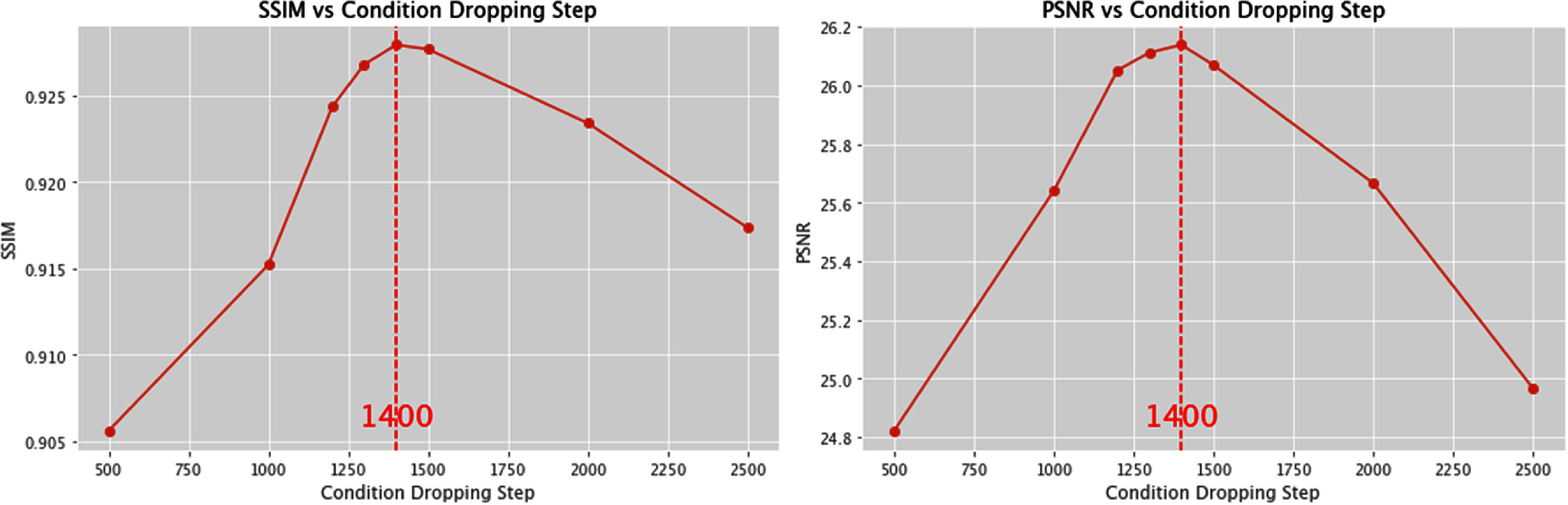
Variations of the LA-CBCT reconstruction quality/accuracy given different condition-dropping steps for PFGDM-A on the test set, with a total reconstruction step of 3000. The limited-angle projections were simulated with orthogonal-view acquisitions for a total scan angle of $30^\circ \left( {\left[ {0^\circ ,15^\circ } \right],\left[ {90^\circ ,105^\circ } \right]} \right),90^\circ \left( {\left[ {0^\circ ,45^\circ } \right],\left[ {90^\circ ,135^\circ } \right]} \right)$, or $120^\circ \left( {\left[ {0^\circ ,60^\circ } \right],\left[ {90^\circ ,150^\circ } \right]} \right)$.

To quantitatively determine the optimal step of dropping the high-frequency information for PFGDM-A, we performed a parameterized study using condition-dropping steps ranging from 500 to 2500. We ran the experiments on the test set with the orthogonal-view acquisitions of limited-angle projections for a total scan angle of $30^\circ ,{\text{ }}90^\circ ,$ and $120^\circ .$ The total reconstruction steps for all experiments are 3000. An experiment with the condition dropping step of 500 means that, from step 0 to step 500 the high-frequency condition is introduced to guide the diffusion model, while from step 501 to step 3000 no condition is used. As shown in figure 4, the optimal condition-dropping step is around 1400, which gives the best SSIM and PSNR results. All reconstructions performed in the following study for PFGDM-A are based on this step number.

### 3.2. Qualitative evaluation

**Figure 5.**
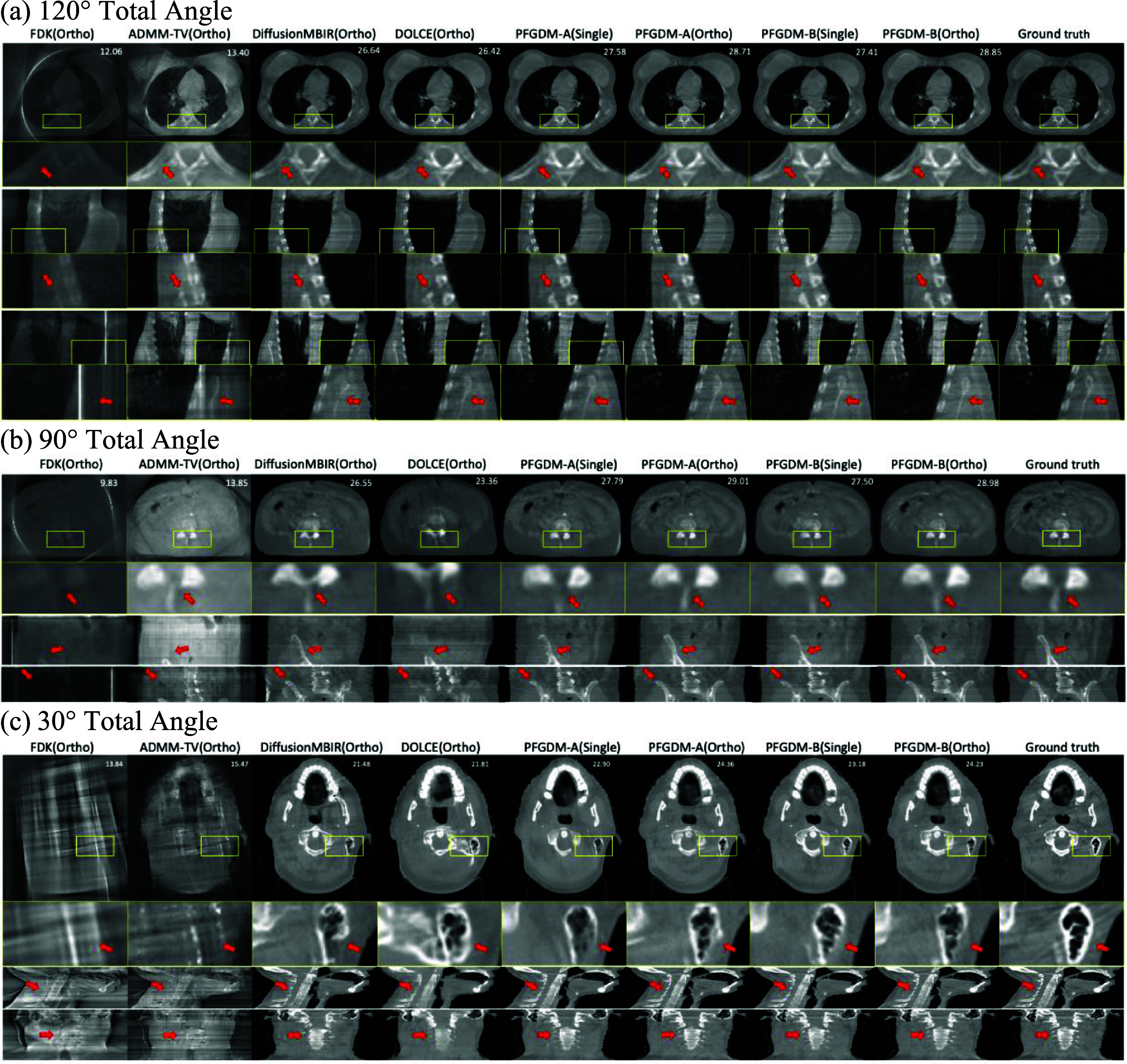
Visual comparison between the reconstructed LA-CBCTs of different methods and the ‘ground truth’. In subfigure (a), first, third, and fifth rows represent axial, coronal, and sagittal views, respectively, and the second, fourth, and sixth rows show a zoomed-in region of the corresponding views. In subfigures (b) and (c), first, third, and fourth rows represent axial, coronal, and sagittal views, respectively, and the second row shows a zoomed-in region of the axial view. The limited-angle projections were simulated with either orthogonal-view (ortho) acquisitions for a total scan angle of $30^\circ \left( {\left[ {0^\circ ,15^\circ } \right],\left[ {90^\circ ,105^\circ } \right]} \right),90^\circ \left( {\left[ {0^\circ ,45^\circ } \right],\left[ {90^\circ ,135^\circ } \right]} \right)$, or $120^\circ \left( {\left[ {0^\circ ,60^\circ } \right],\left[ {90^\circ ,150^\circ } \right]} \right);$ or single-view (single) acquisitions for a total scan angle of $30^\circ \left( {\left[ {0^\circ ,30^\circ } \right]} \right),90^\circ \left( {\left[ {0^\circ ,90^\circ } \right]} \right),$ or $120^\circ \left( {\left[ {0^\circ ,120^\circ } \right]} \right)$. Three scenarios of different total scan angles and anatomies are shown as panel (a)–(c) respectively. PSNR scores of each reconstruction on axial slices against the ‘ground truth’ are indicated at the top-right corner of each figure. See table 1 for quantitative results.

We visually compared the reconstructed LA-CBCT images between FDK, ADMM-TV, DOLCE, DiffusionMBIR, PFGDM-A, and PFGDM-B in figure 5. Different panels show the results of different scan angles and anatomical sites. The LA-CBCTs reconstructed by the conventional FDK algorithm were compromised by strong under-sampling artifacts, while the iterative ADMM algorithm with TV regularization (ADMM-TV) manages to substantially suppress these artifacts but unable to reconstruct the anatomical details, especially for relatively small scan angles (30°). With diffusion models introduced as denoisers, DiffusionMBIR and DOLCE outperformed ADMM-TV, while the reconstructed anatomical details showed clear mismatches with the ‘ground truth’, especially for the 30° scan angle. In contrast, with the high-frequency prior CT condition and condition phasing-out schemes, PFGDM-A and PFGDM-B reconstructed the anatomical structures to match well with the ‘ground truth’ for axial, coronal, and sagittal views, even for the 30° scan angle scenario. Their reconstruction results using single-view projections are generally better than those of DiffusionMBIR and DOLCE, even though DiffusionMBIR and DOLCE used the more efficient orthogonal-view sampling. PFGDM-A and PFGDM-B showed comparable performance under both single-view and orthogonal-view sampling conditions.

### 3.3. Quantitative evaluation

Table 1 quantitatively compares different methods on their reconstruction accuracy. Most methods used the orthogonal-view scheme (ortho), while PFGDM-A and PFGDM-B used both single-view (single) and orthogonal-view (ortho) sampling scenarios. As shown, PFGDM-A and PFGDM-B consistently outperformed other methods under all limited-angle scenarios. The advantage of PFGDM becomes especially evident when the scan angle decreases, showing the efficacy of introducing prior CT information when the on-board projection information is limited. For 90- and 120-degree reconstruction, PFGDM-B performed slightly better than PFGDM-A. Notably, PFGDM-A and PFGDM-B based on single-view projections already yielded better or comparable results when compared with other methods that were based on orthogonal-view projections.

**Table 1.** Average (±s.d.) PSNR and SSIM results on the test set by different methods, on axial slices. The arrows are pointing in the direction of improved accuracy. **Best values** and second-best values for each metric are color-coded.

Metric/Angle	PSNR↑			SSIM↑		
	30°	90°	120°	30°	90°	120°
FDK (Ortho)	12.66 ± 1.12	13.96 ± 1.06	14.83 ± 1.74	0.304 ± 0.115	0.431 ± 0.201	0.511 ± 0.201
ADMM-TV (Ortho)	12.86 ± 1.74	14.18 ± 2.99	15.59 ± 3.87	0.533 ± 0.072	0.688 ± 0.044	0.773 ± 0.068
DOLCE (Ortho)	18.57 ± 2.53	22.74 ± 3.82	25.51 ± 3.03	0.776 ± 0.068	0.868 ± 0.096	0.896 ± 0.106
DiffusionMBIR (Ortho)	19.61 ± 2.47	24.58 ± 4.18	26.18 ± 4.89	0.807 ± 0.048	0.912 ± 0.068	0.926 ± 0.067
PFGDM-B (Single)	22.05 ± 0.85	25.48 ± 1.01	27.22 ± 0.88	0.851 ± 0.054	0.926 ± 0.021	0.946 ± 0.013
PFGDM-B (Ortho)	23.72 ± 1.19	**26.68 ± 1.04**	**28.20 ± 1.28**	0.894 ± 0.034	**0.941 ± 0.014**	**0.954 ± 0.011**
PFGDM-A (Single)	22.21 ± 1.16	25.80 ± 1.28	27.77 ± 1.42	0.855 ± 0.055	0.929 ± 0.021	0.949 ± 0.013
PFGDM-A (Ortho)	**23.81 ± 2.25**	26.63 ± 2.79	27.97 ± 3.10	**0.896 ± 0.036**	0.937 ± 0.029	0.949 ± 0.027

### 3.4. Extreme cases

**Figure 6.**
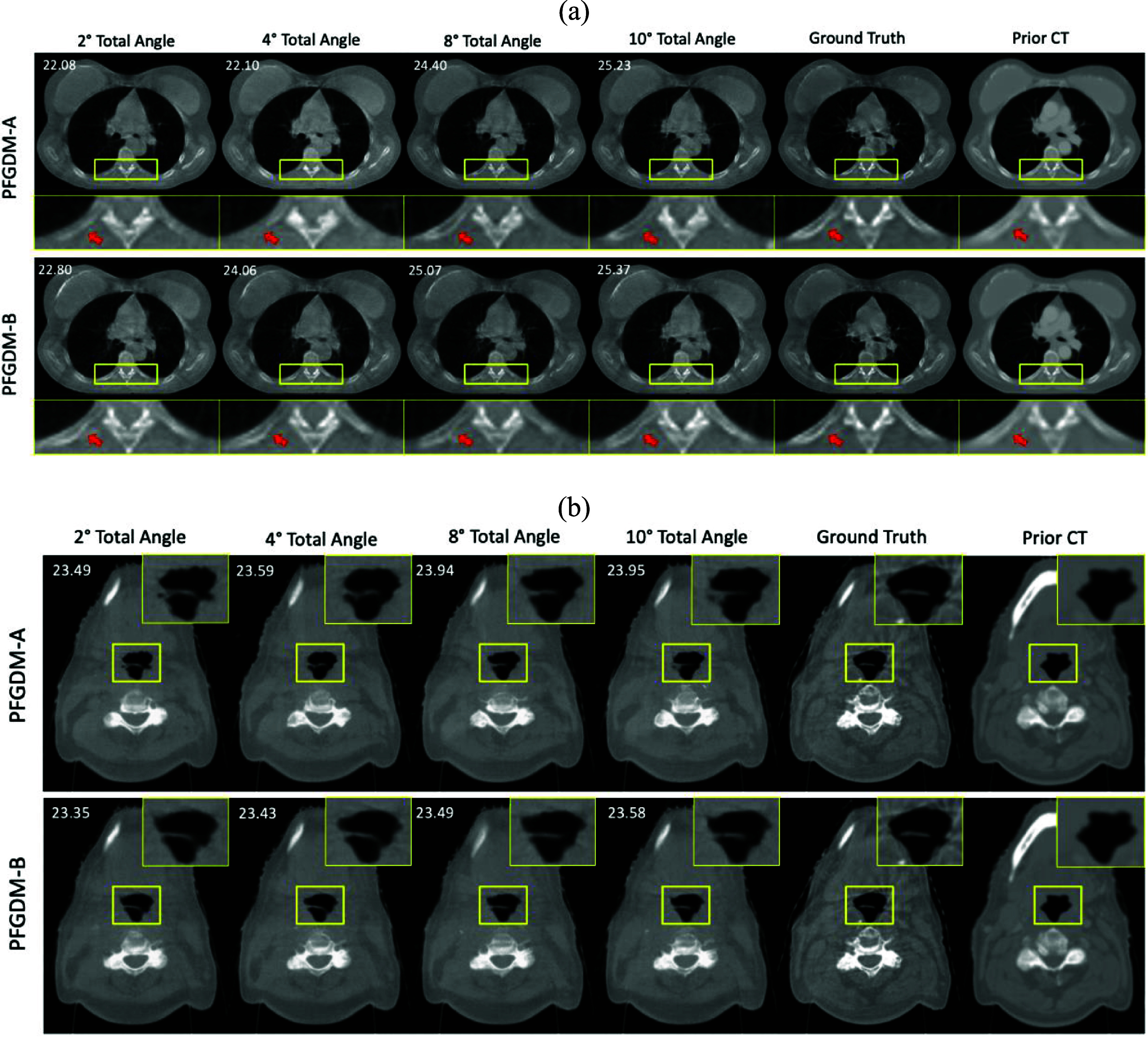
Performance of PFGDM-A and PFGDM-B under extremely limited scan angle scenarios. The limited-angle projections were simulated with orthogonal-view acquisitions for a total scan angle of 2$^\circ \left( {\left[ {0^\circ ,1^\circ } \right],\left[ {90^\circ ,91^\circ } \right]} \right),4^\circ \left( {\left[ {0^\circ ,2^\circ } \right],\left[ {90^\circ ,92^\circ } \right]} \right)$, 8$^\circ \left( {\left[ {0^\circ ,4^\circ } \right],\left[ {90^\circ ,94^\circ } \right]} \right),$ or 10$^\circ \left( {\left[ {0^\circ ,5^\circ } \right],\left[ {90^\circ ,95^\circ } \right]} \right)$. PSNR scores of each reconstruction against the ‘ground truth’ are indicated at the top-left corner of each figure. See table 2 for quantitative results.

The visual comparison of the reconstructed LA-CBCTs of PFGDM-A and PFGDM-B using extremely limited scan angles is shown in figure 6. In figure 6(a), PFGDM-B performed consistently better than PFGDM-A on this test sample with higher PSNR scores. PFGDM-B preserved the anatomical details of bony structures even though very limited information from CBCT projections is available. The slightly inferior performance of PFGDM-A could be due to the fact that the condition dropping step was optimized based on a different set of scan angles (figure 4). In figure 6(b), PFGDM-A and PFGDM-B showed very similar performance. Even though the anatomical structures of the zoomed-in region showed clear differences between prior CT and ‘ground-truth’ CBCT, both of the PFGDM variants produced high-fidelity reconstructions of LA-CBCT. Overall, as shown in table 2, PFGDM-A and PFGDM-B had similar SSIM and PSNR scores.

**Table 2.** Average (±s.d.) PSNR and SSIM results of PFGDM-A and PFGDM-B under extreme limited-angle scenarios, on axial slices. The arrows are pointing in the direction of improved accuracy. **Bold**: Best values.

Metric/Angle	PSNR↑				SSIM↑			
	2°	4°	8°	10°	2°	4°	8°	10°
PFGDM-B (Ortho)	**22.03 ± 1.25**	**22.23 ± 1.33**	22.52 ± 1.34	22.63 ± 1.31	0.849 ± 0.051	0.854 ± 0.051	0.864 ± 0.047	0.867 ± 0.045
PFGDM-A (Ortho)	21.97 ± 1.68	22.19 ± 1.81	**22.53 ± 1.91**	**22.71 ± 1.98**	**0.851 ± 0.049**	**0.857 ± 0.046**	**0.865 ± 0.044**	**0.869 ± 0.044**

### 3.5. Noise study

**Figure 7.**
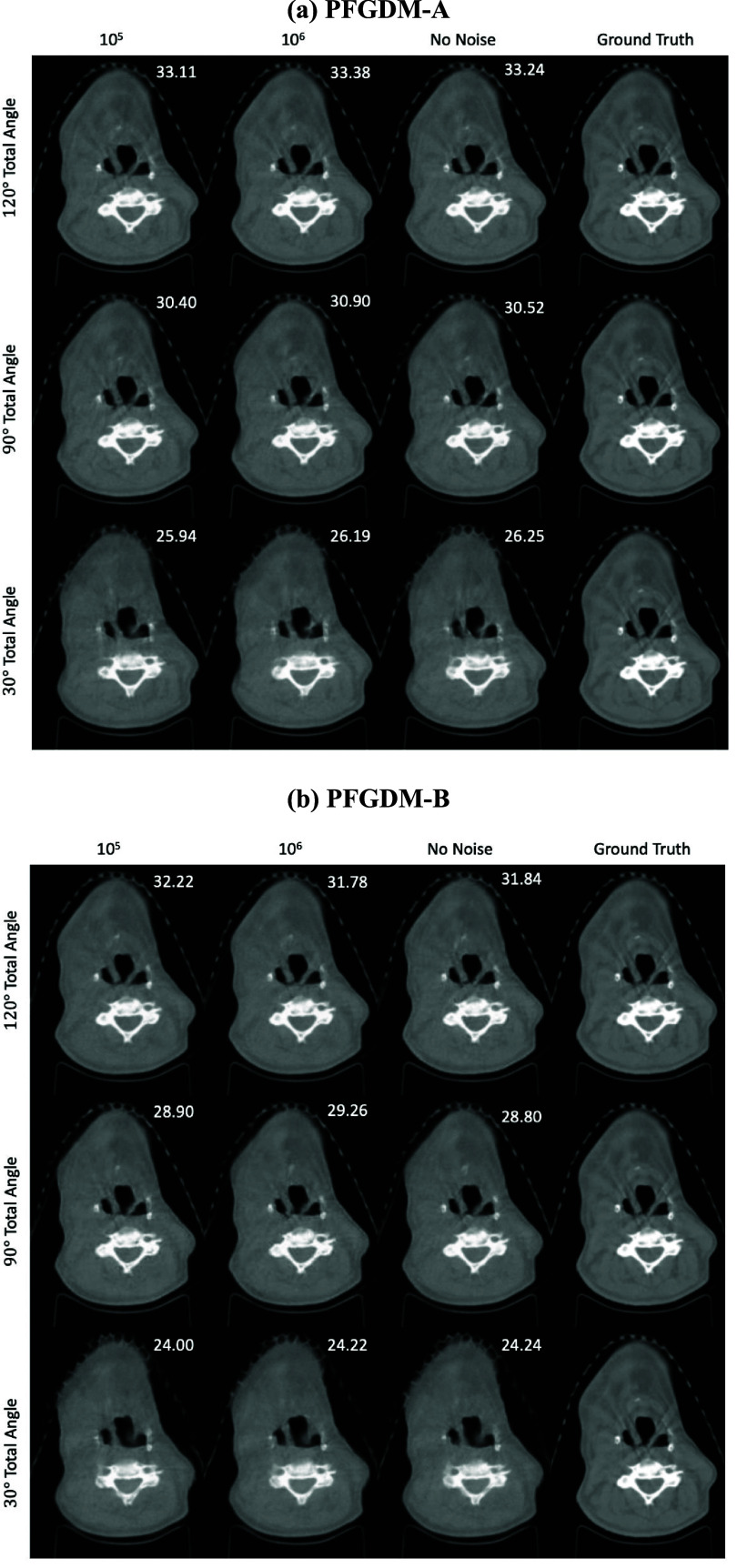
Performance of PFGDM-A and PFGDM-B under noise-augmented projections. 10^5^ and 10^6^ indicate the photon number per pixel used for noise simulation, with a smaller photon number corresponding to a higher noise level. The limited-angle projections were simulated with either orthogonal-view (ortho) acquisitions for a total scan angle of 30°([0°,15°],[90°,105°]), 90°([0°,45°],[90°,135°]), or 120°([0°,60°],[90°,150°]). PSNR scores of each reconstruction against the ‘ground truth’ are indicated at the top-right corner of each figure. See table 3 for quantitative results.

The visual comparison of the reconstructed LA-CBCTs by PFGDM-A and PFGDM-B, using projections augmented with different levels of noise, is shown in figure 7. Albeit some fluctuations, more noise (fewer photon counts) yields less accurate results, although the difference is very small. In the case with ${10^5}$ photon counts per pixel (which is considered a normal noise level), the results are stable, indicating a limited impact of noise on the reconstruction accuracy.

### 3.6. Impacts of misaligned prior frequency

**Figure 8.**
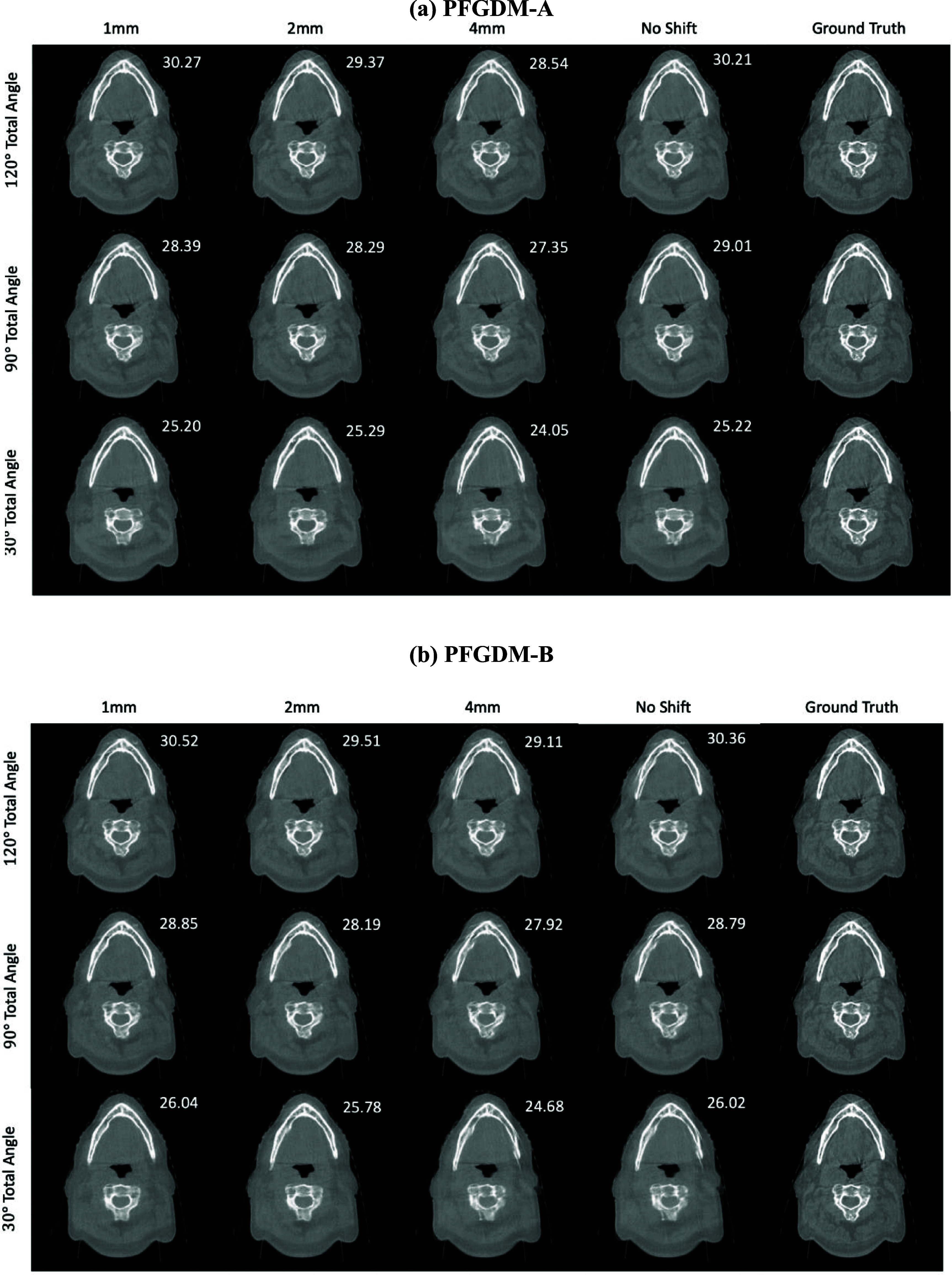
Performance of PFGDM-A and PFGDM-B under scenarios of prior frequency guidance misaligned with the ‘ground-truth’ CBCT. 1 mm, 2 mm, and 4 mm indicate the misalignment magnitudes. The limited-angle projections were simulated with either orthogonal-view (ortho) acquisitions for a total scan angle of 30°([0°,15°],[90°,105°]), 90°([0°,45°],[90°,135°]), or 120°([0°,60°],[90°,150°]). PSNR scores of each reconstruction against the ‘ground truth’ are indicated at the top-right corner of each figure. See table 4 for quantitative results.

The visual comparison of the reconstructed LA-CBCTs by PFGDM-A and PFGDM-B using different magnitudes of shifted CT priors is shown in figure 8. Compared with using the registered CT prior, PFGDM-A and PFGDM-B generate similar CBCT reconstruction results when the CT priors are shifted by 1 mm and 2 mm. When the mismatch increases to 4 mm, the degradations become more pronounced. PFGDM is shown to be resilient to small registration errors between the to-be-reconstructed CBCT and the prior CT, with the required accuracy achievable by clinical 2D x-ray matching or 2D/3D registration. It is notable that the prior CT was rigidly registered to the reference CBCT in our experiment, and there could exist additional registration errors from this rigid registration, indicating PFGDM can be more tolerant to the misalignment than assumed.

### 3.7. Ablation studies

#### 3.7.1. No prior reconstruction

**Figure 9.**
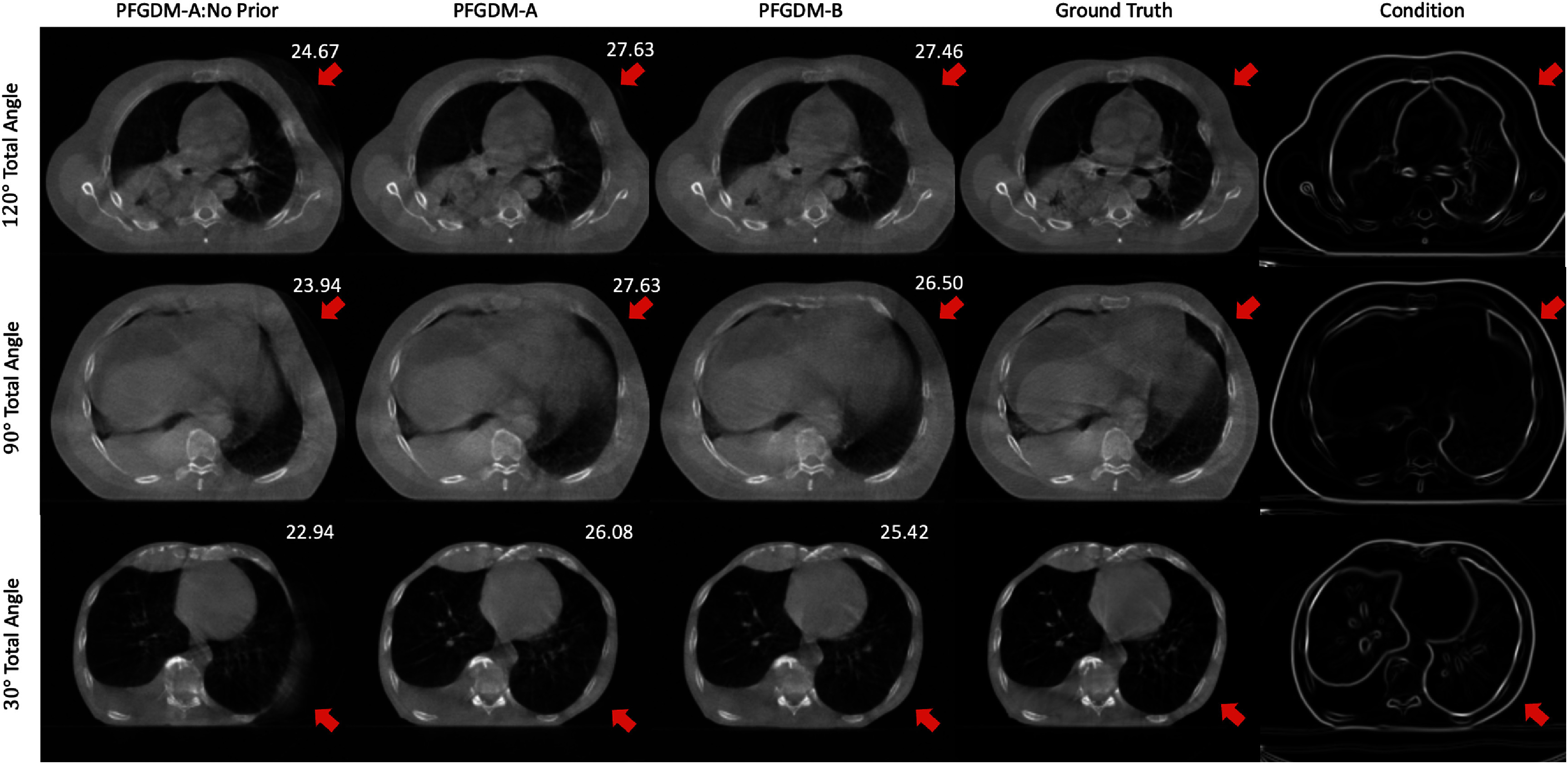
Visual comparisons of the reconstructed LA-CBCTs between PFGDM-A with no prior information (condition dropping step equals to 1), PFGDM-A with a condition dropping step of 1400, PFGDM-B, and the ‘ground-truth’ CBCT. The limited-angle projections were simulated with either orthogonal-view (ortho) acquisitions for a total scan angle of 30°([0°,15°],[90°,105°]), 90°([0°,45°],[90°,135°]), or 120°([0°,60°],[90°,150°]). PSNR scores of each reconstruction on axial slices against the ‘ground truth’ are indicated at the top-right corner of each figure. See table 5 for quantitative results.

The visual comparison of the reconstructed LA-CBCTs without the prior high-frequency condition is shown in figure 9. It can be seen that without the guidance of the prior information, the model can reconstruct most of the anatomical structures of CBCT. However, it fails to retain some of the structure as pointed out by red arrows in figure 9 due to the limited-angle acquisitions. Thus, the high-frequency prior of our proposed PFGDM framework plays a crucial part in achieving optimal performance.

#### 3.7.2. Prior frequency condition-only reconstruction

**Figure 10.**
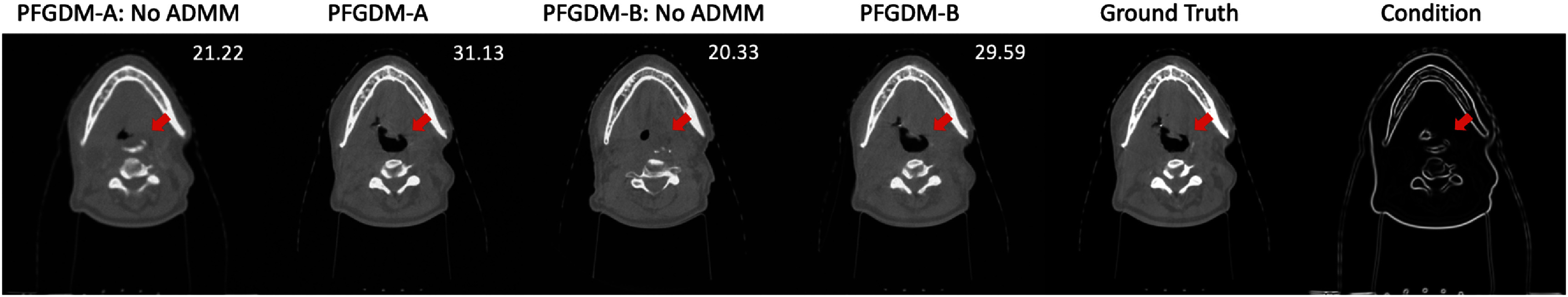
Visual results on the reconstructed LA-CBCTs by removing the ADMM step. The limited-angle projections were simulated with orthogonal-view acquisitions for a total scan angle of $90^\circ \left( {\left[ {0^\circ ,45^\circ } \right],\left[ {90^\circ ,135^\circ } \right]} \right)$. PSNR scores of each reconstruction on axial slices against the ‘ground truth’ are indicated at the top-right corner of each figure. See table 5 for quantitative results.

The visual comparison of the reconstructed LA-CBCTs by PFGDM-A and PFGDM-B conditioned on prior CT information only (without ADMM) is shown in figure 10. As expected, the prior CT information guidance helps to generate a CBCT very similar to the CT image. However, without the limited-angle reconstruction from the ADMM step, both PFGDM-A and PFGDM-B fail to reconstruct the anatomical differences pointed out by red arrows in figure 10 between the ‘ground-truth’ CBCT and the prior CT condition. In contrast, our proposed PFGDM methods with ADMM successfully reconstructed the mismatches with much higher PSNR scores. Therefore, the ADMM step serves as a necessary corrector to reconstruct the anatomical differences from limited-angle projections, which the diffusion model cannot fulfill.

**Table 3.** Average (±s.d.) PSNR and SSIM results of PFGDM-A and PFGDM-B with different levels of simulated projection noise. 10^5^ and 10^6^ indicate the photon number per pixel used for noise simulation, with a smaller photon number corresponding to a higher noise level. The arrows are pointing in the direction of improved accuracy.

Noise level/Angle		10^5^		10^6^		No Noise	
	Metric	PSNR↑	SSIM↑	PSNR↑	SSIM↑	PSNR↑	SSIM↑
30°	PFGDM-B	23.11 ± 2.28	0.875 ± 0.054	23.62 ± 1.33	0.889 ± 0.037	23.72 ± 1.19	0.894 ± 0.034
	PFGDM-A	23.02 ± 1.94	0.863 ± 0.052	23.78 ± 1.17	0.901 ± 0.031	23.81 ± 2.25	0.896 ± 0.036
90°	PFGDM-B	26.21 ± 2.53	0.921 ± 0.058	26.67 ± 1.18	0.939 ± 0.016	26.68 ± 1.04	0.941 ± 0.014
	PFGDM-A	26.51 ± 2.82	0.931 ± 0.030	26.74 ± 1.16	0.944 ± 0.013	26.63 ± 2.79	0.937 ± 0.029
120°	PFGDM-B	28.02 ± 4.03	0.944 ± 0.061	28.08 ± 1.44	0.953 ± 0.012	28.20 ± 1.28	0.954 ± 0.011
	PFGDM-A	28.01 ± 1.23	0.951 ± 0.011	27.93 ± 3.25	0.947 ± 0.031	27.97 ± 3.10	0.949 ± 0.027

## 4. Discussion

CBCT plays a crucial role in image-guided radiotherapy, and there is a continuing interest in reconstructing CBCTs from limited-angle acquisitions (LA-CBCT) to enhance imaging efficiency, reduce radiation exposure, and improve mechanical clearance. LA-CBCT reconstruction, however, suffers from severe under-sampling artifacts and distortions, making it a highly ill-posed inverse problem. In this study, we proposed a diffusion model-based framework, PFGDM, to address the LA-CBCT reconstruction problem. PFGDM uses high-frequency information from patients’ prior CT scans as anatomical detail-preserving conditions to feed into a diffusion model-based regularizer, which was incorporated into an iterative reconstruction framework based on the ADMMs. The two implementations of this framework, PFGDM-A and PFGDM-B, yielded similar reconstruction results that were close to fully-sampled reconstructions, and outperformed standard FDK, regularized iterative reconstruction (ADMM-TV), and current SOTA diffusion network-based reconstructing approaches including DiffusionMBIR and DOLCE (figure 5 and table 1).

**Figure 11.**
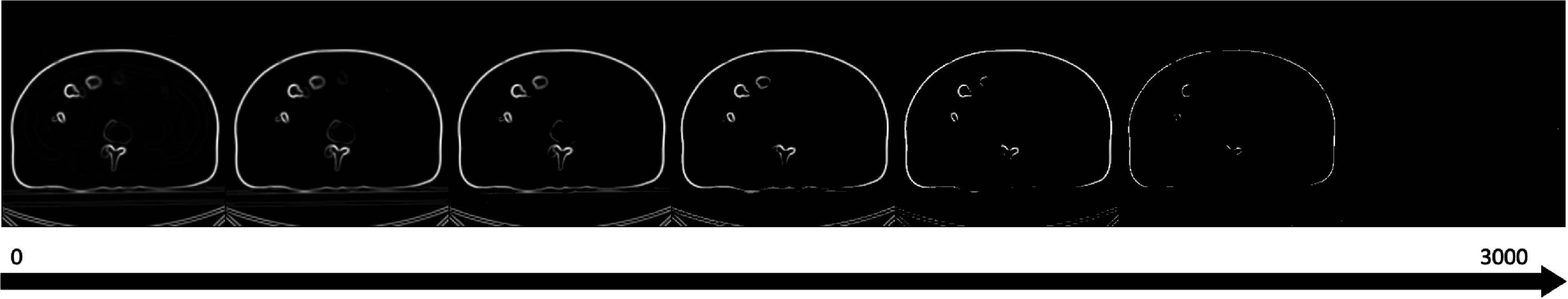
Sample conditional input of PFGDM-B from reconstruction step 0 to step 3000, with a step-interval of 500.

**Figure 12.**
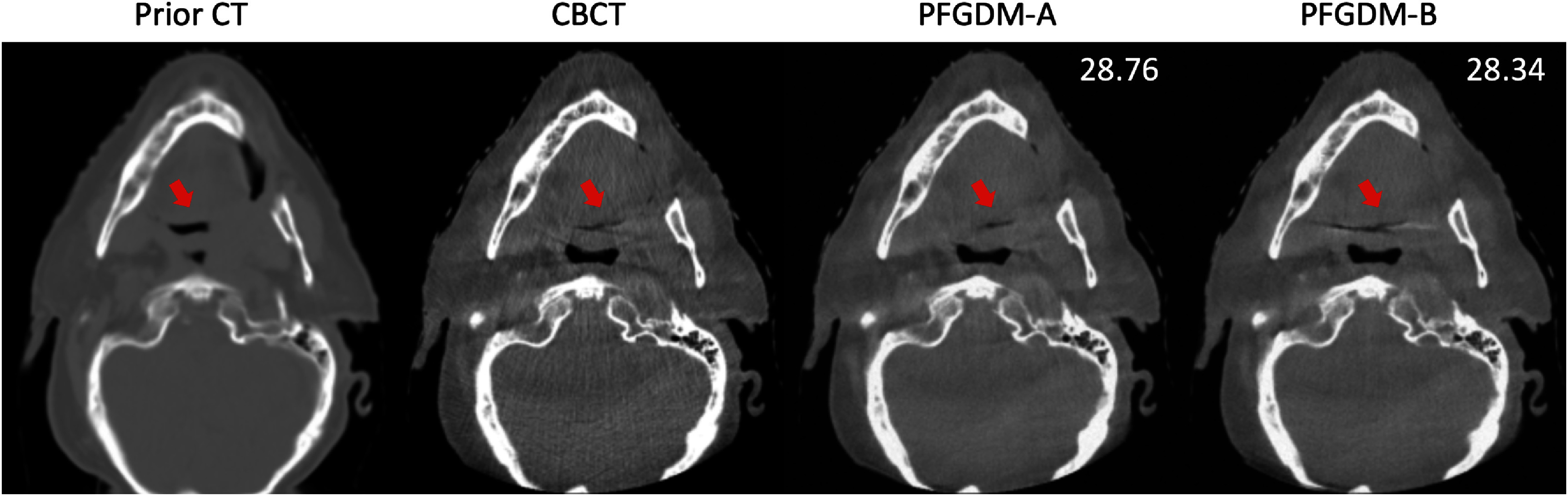
PFGDM-A and PFGDM-B reconstruction results when CT/CBCT have clear anatomical differences. The limited-angle projections were simulated by the orthogonal-view geometry for a total scan angle of $120^\circ \left( {\left[ {0^\circ ,60^\circ } \right],\left[ {90^\circ ,150^\circ } \right]} \right)$. PSNR scores of each reconstruction against the ‘ground-truth’ CBCT are indicated at the top-right corner.

Of the two PFGDM variants, PFGDM-A incorporates the same conditional input from the high-frequency information of the patient’s prior CT until a pre-defined step. Our parametric study (figure 4) shows that discarding the conditional information at an intermediate step leads to better results, which stems from the anatomical changes between the planning CT and the daily CBCT (figure 3). Per our study, stopping introducing high-frequency conditions after 1400 reconstruction steps achieves the optimal result in general (figure 4). However, such an optimized number may change across different datasets or scanning conditions. Given a new data distribution, a parametric study may be needed to find the optimal condition dropping step. PFGDM-B, on the other hand, circumvents this potential issue by dynamically changing the conditional input during the entire reconstruction process. The high-frequency information corresponding to CT anatomical boundaries is gradually phased out to prevent the model from being overly influenced by the prior CT information (figure 11). In our experiments, the qualitative and quantitative results of PFGDM-A and PFGDM-B are comparable. However, in a few scenarios where CT and CBCT present clear anatomical differences, PFGDM-A was shown to provide better performance than PFGDM-B, as the latter appeared more impacted by the incorrect condition (figure 12). More investigations are warranted in future studies to investigate the correlation between the condition’s accuracy and the LA-CBCT reconstruction accuracy, as well as its impacts on the two PFGDM variants.

**Table 4.** Average (±s.d.) PSNR and SSIM results of PFGDM-A and PFGDM-B under scenarios of prior frequency guidance misaligned with the ‘ground-truth’ CBCT. 1 mm, 2 mm, and 4 mm indicate the misalignment magnitudes. The arrows are pointing in the direction of improved accuracy.

Shift amount		1 mm		2 mm		4 mm		No Shift	
/Angle	Metric	PSNR↑	SSIM↑	PSNR↑	SSIM↑	PSNR↑	SSIM↑	PSNR↑	SSIM↑
30°	PFGDM-B	23.72 ± 1.18	0.894 ± 0.035	23.44 ± 1.21	0.891 ± 0.036	22.33 ± 2.71	0.865 ± 0.062	23.72 ± 1.19	0.894 ± 0.034
	PFGDM-A	23.78 ± 2.24	0.895 ± 0.037	23.68 ± 2.26	0.894 ± 0.036	23.48 ± 2.36	0.884 ± 0.044	23.81 ± 2.25	0.896 ± 0.036
90°	PFGDM-B	26.64 ± 1.11	0.941 ± 0.015	26.51 ± 1.08	0.939 ± 0.015	26.16 ± 1.12	0.935 ± 0.018	26.68 ± 1.04	0.941 ± 0.014
	PFGDM-A	26.58 ± 2.79	0.936 ± 0.029	26.53 ± 2.82	0.936 ± 0.031	26.30 ± 2.93	0.933 ± 0.034	26.63 ± 2.79	0.937 ± 0.029
120°	PFGDM-B	28.16 ± 1.31	0.953 ± 0.012	28.11 ± 1.24	0.953 ± 0.011	27.87 ± 1.22	0.951 ± 0.012	28.20 ± 1.28	0.954 ± 0.011
	PFGDM-A	27.94 ± 3.14	0.948 ± 0.027	27.92 ± 3.15	0.948 ± 0.028	27.77 ± 3.20	0.946 ± 0.031	27.97 ± 3.10	0.949 ± 0.027

**Table 5.** Average (±s.d.) PSNR and SSIM results of PFGDM without the prior CT guidance or without the ADMM updating step. Note that the projection scan angle becomes irrelevant when the ADMM updating step is removed. The arrows are pointing in the direction of improved accuracy. **Bold**: Best values.

Metric/Angle	PSNR↑			SSIM↑		
	30°	90°	120°	30°	90°	120°
PFGDM-B (Ortho)	23.72 ± 1.19	**26.68 ± 1.04**	**28.20 ± 1.28**	0.894 ± 0.034	**0.941 ± 0.014**	**0.954 ± 0.011**
PFGDM-A (Ortho)	**23.81 ± 2.25**	26.63 ± 2.79	27.97 ± 3.10	**0.896 ± 0.036**	0.937 ± 0.029	0.949 ± 0.027
PFGDM-A (No prior)	19.57 ± 1.49	24.68 ± 1.44	26.65 ± 2.08	0.851 ± 0.057	0.927 ± 0.021	0.934 ± 0.013
PFGDM-B (No ADMM)		17.67 ± 1.98			0.712 ± 0.122	
PFGDM-A (No ADMM)		18.43 ± 1.27			0.731 ± 0.151	

The framework of PFGDM is built upon the assumption that patient-specific prior CTs share a similar underlying patient anatomy with the CBCTs. The assumption is generally valid although there exist various degrees of anatomical mismatches due to patient setup variations, anatomical deformations, treatment responses, or patient on-board motion. In this study, we pre-processed the CT and CBCT images by rigidly registering them, such that the impact of rigid patient setup variations can be reduced. In real clinical practices, a rigid setup correction can be performed to serve the purpose via 2D radiography-based matching, surface image-guided matching, or 2D/3D registration techniques. However, the remaining deformation between CT and CBCT cannot be corrected and relies on the PFGDM to further reconstruct the anatomical differences. We tested the performance of PFGDM to the shifted CT priors and found that it is resilient to minor registration mismatches between CBCT-CT. The results showed that relatively small registration errors have limited impacts on the PFGDM accuracy, although some degradations can be observed for the 4 mm shift scenarios. Thus, perfect registration is not required for PFGDM to work and manual clinical alignment using x-ray and digitally reconstructed radiograph pairs may suffice. To alleviate the burden on PFGDM to reconstruct all the deformation-induced anatomical variations, we can potentially apply 2D–3D deformable registration (Zhang 2021) to bring the prior CT closer to the to-be-solved CBCT, which may potentially further improve the accuracy of PFGDM. Alternatively, we can also use a fully-sampled CBCT volume acquired in previous treatment fractions to serve as the prior, which may contain a patient anatomy closer to the to-be-reconstructed CBCT and improve the overall reconstruction accuracy.

Our study further supported the finding that the orthogonal-view geometry yields better reconstruction results than the single-view geometry, even if the total scan angle remains the same. Therefore, orthogonal-view projections are preferred in limited-angle CBCT reconstruction tasks. In our study the orthogonal-view projections were simulated in kilovoltage (kV) energy for both directions. However, simultaneous orthogonal-view kV–kV acquisitions are currently not available on clinical radiotherapy devices and limited to experimental setups only (Ren *et al* 2016). With orthogonally-arranged kV and megavoltage (MV) imaging systems equipped on most modern LINACs, orthogonal-view kV–MV projections are available for PFGDM reconstruction. Previous studies have attempted to harmonize the intensity differences between the kV and MV projections by applying pixel intensity linear scaling or using normalized cross correlation as the similarity metric (Ren *et al* 2014, Zhang *et al* 2017b), which however requires further investigations for potential translations into PFGDM reconstruction.

The study shows the feasibility of reconstructing CBCT images from limited-angle 2D x-ray projections guided by a conditional diffusion model. However, several limitations need to be considered and addressed before potential clinical applications. First, the reconstruction of PFGDM is time consuming, which takes around 3 h to reconstruct a CBCT volume of 80 slices. The low efficiency is mainly caused by the slow diffusion models. To improve the efficiency, the current score-based diffusion model can be replaced by the denoising diffusion implicit model (Song and Ermon 2020) which allows step-skipping in the denoising process for acceleration. In addition, further parallelization of the ADMM optimization can also boost the reconstruction speed (Deng *et al* 2017). Second, the cone-beam x-ray projections were simulated in our experiments from CBCT images. Further evaluation of PFGDM on real cone-beam projections is warranted. Third, we used fully-sampled FDK CBCTs from the clinic as the ‘ground truth’ in this study. However, these images still contain artifacts (Schulze *et al* 2011) that compromise the fidelity of the ‘ground truth’, leading to potential inaccuracies in quantitative analysis. As an alternative, techniques such as iterative reconstructions (Sun *et al* 2015) and DL-based methods (Bayaraa *et al* 2020, Amirian *et al* 2024) have shown capabilities in enhancing CBCT quality and reducing artifacts, which can potentially be used to reconstruct the CBCTs to serve as the ‘ground truth’, if the raw x-ray projections are available.

## 5. Conclusion

In this work, we introduced PFGDM, a diffusion model-based method for LA-CBCT reconstruction. PFGDM incorporates the high-frequency information from the patient’s existing CT scans as additional priors to compensate the missing angle information in LA-CBCT reconstruction. Within this framework, we further developed two variants, PFGDM-A and PFGDM-B, to evaluate different condition phasing-out strategies to allow the PFGDM to better reconstruct CT/CBCT differences. Both variants of PFGDM outperformed the other LA-CBCT reconstruction techniques, especially for relatively small scan angles. The tests on extremely limited scan angles also demonstrated the potential of PFGDM-based methods. PFGDM makes a general method that does not need re-training for different imaging geometries or limited-angle scenarios, providing it a substantial advantage over methods like DOLCE for potential clinical translation.

## Data Availability

The data cannot be made publicly available upon publication because they contain sensitive personal information. The data that support the findings of this study are available upon reasonable request from the authors.
